# Correction: Demineralized Bone Matrix Combined Bone Marrow Mesenchymal Stem Cells, Bone Morphogenetic Protein-2 and Transforming Growth Factor-β3 Gene Promoted Pig Cartilage Defect Repair

**DOI:** 10.1371/journal.pone.0125948

**Published:** 2015-05-13

**Authors:** Xin Wang, Yanlin Li, Rui Han, Chuan He, Guoliang Wang, Jianwei Wang, Jiali Zheng, Ming Pei, Lei Wei

The eighth’s author name is spelled incorrectly. The correct name is: Ming Pei.
